# Prevalence of Acute Respiratory Infections in Women and Children in Western Sierra Leone due to Smoke from Wood and Charcoal Stoves

**DOI:** 10.3390/ijerph9062252

**Published:** 2012-06-19

**Authors:** Eldred Tunde Taylor, Satoshi Nakai

**Affiliations:** 1 Graduate School of Environment and Information Sciences, Yokohama National University, 79-7 Tokiwadai, Hodogaya-ku, Yokohama, Kanagawa 240-8501, Japan; Email: snakai@ynu.ac.jp; 2 Institute of Environmental Management and Quality Control, Njala University, Njala, Moyamba District, Sierra Leone

**Keywords:** cooking fuel, acute respiratory infections, suspended particulate matter

## Abstract

Combustion of biomass fuels (wood and charcoal) for cooking releases smoke that contains health damaging pollutants. Women and children are the most affected. Exposure to biomass smoke is associated with acute respiratory infections (ARI). This study investigated the prevalence of ARI potentially caused by smoke from wood and charcoal stoves in Western Sierra Leone, as these two fuels are the predominant fuel types used for cooking. A cross sectional study was conducted for 520 women age 15–45 years; and 520 children under 5 years of age in homes that burn wood and charcoal. A questionnaire assessing demographic, household and exposure characteristics and ARI was administered to every woman who further gave information for the child. Suspended particulate matter (SPM) was continuously monitored in fifteen homes. ARI prevalence revealed 32% and 24% for women, 64% and 44% for children in homes with wood and charcoal stoves, respectively. After adjusting for potential confounders for each group, the odds ratio of having suffered from ARI was similar for women, but remained large for children in homes with wood stoves relative to charcoal stoves (OR = 1.14, 95%CI: 0.71–1.82) and (OR = 2.03, 95%CI: 1.31–3.13), respectively. ARI prevalence was higher for children in homes with wood stoves compared with homes with charcoal stoves, but ARI prevalence for both types of fuels is higher compared with reported prevalence elsewhere. To achieve a reduction in ARI would require switching from wood and charcoal to cleaner fuels.

## 1. Introduction

It has been estimated that approximately three billion people worldwide rely on biomass fuels (wood, charcoal, animal dung, crop residues, *etc.*) for domestic cooking [1]. These fuels are at the bottom of the energy ladder, and are classified as crude/dirty fuels [2]. They are burnt indoors in open fires or poorly functioning stoves, often causing high levels of pollution. Smoke released from burning biomass fuels is a complex mixture of pollutants that comprises particulate matter, carbon monoxide, nitrogen dioxide, volatile organic compounds, aromatic hydrocarbons, *etc*. [3]. Women and children receive the highest exposure of biomass smoke in developing countries [4], because of their frequent presence in the kitchen environment. 

Exposure to biomass smoke is a major health risk, particularly in developing countries. It is well documented that exposure to biomass smoke is associated with acute respiratory infections (ARI) in children [5], and ARI, tuberculosis, lung cancer and chronic obstructive pulmonary disease (COPD) in adults [6,7,8,9,10]. According to a recent estimate, acute lower respiratory infection is the leading cause of global mortality mostly in developing countries [11]. The African region has, in general, the highest burden of childhood mortality, with 45% of global under-5 deaths from pneumonia. A number of studies have examined the association between smoke and some of its constituents derived from burning biomass and ARI in children of developing countries [12,13,14,15,16]. Some of these authors used the type of cooking fuel to group children into categories of exposure [14,15]. They used children from homes using cleaner fuels such as liquefied petroleum gas or electricity as a reference group to determine the health effects caused by biomass smoke. 

In Sierra Leone, only about 5% of the populace is reported to use any form of clean fuel [17]. This implies that about 95% of people in Sierra Leone depend on crude fuels, mainly wood and charcoal. The majority of rural dwellers depend almost exclusively on wood [18], so it is difficult to conduct health studies based on comparing homes that use biomass fuels relative to those using cleaner fuels, given the prevailing energy consumption patterns. As you transition from rural communities to peri-urban areas, people tend to use a mixture of wood and charcoal, while in urban communities charcoal is predominant. The present study was conducted in rural and peri-urban communities in Western Sierra Leone where households depend typically on wood and charcoal fuels. These fuels are noted to generate substantial emissions, but at varying rates. For instance, burning charcoal fuels emit less particulate matter than burning wood [19,20]. According to Ellegard, the mean emission levels of PM_10_ for wood and charcoal stoves during cooking were 1,220 and 540 µg/m^3^, respectively [20]. Thus, even though these fuels are both polluting in nature, their relative effects in terms of ARI might be different. To the best of our knowledge, most studies do not directly compare these two fuels, but such information is important to understand wood- and charcoal-related ARI issues in typical communities that depend on these fuels. 

This study therefore investigated the prevalence of ARI potentially caused by smoke derived from wood and charcoal stoves, to test the hypothesis that women and children living in homes with wood stoves have more ARI prevalence than those using charcoal. We further assessed the levels of suspended particulate matter (SPM) in selected households using these fuels to ascertain the levels in homes with reported ARI cases. 

## 2. Methods

Sierra Leone, with an approximate population of 5.4 million, lies along the West African coastline, and around one fourth of the population resides in the Western Area. The study was conducted in Western Sierra Leone in areas outside Freetown, the capital city. The altitude ranges from 20–150 m above sea level, and the climate, as in most West African countries, is tropical, with average temperature and humidity of 26 °C and 80%, respectively. 

### 2.1. Health Data Collection

This study used a cross sectional survey by administering a questionnaire designed for women and children. A stratified sampling method was used to collect samples. Field volunteers assisted in identifying women with at least one child in homes using wood and charcoal stoves in Western Sierra Leone. Sixteen strata were identified with various population sizes. The number of subjects in each community (stratum) who consented to participate in the survey varied from 23 to 50 based on the population size of that community. They were randomly selected in each stratum from among all women with at least a child in communities that stretched along rural to peri-urban areas (Kent-Lower Allen Town) in Western Sierra Leone. The survey collected information from 520 women age 15–45 years, and the same number of children age 0–59 months. The study was conducted in September 2011 (rainy season). 

The questionnaire that was used to collect data during the survey has been described elsewhere [14]. It contained demographic and health information for both women and children. The variables for women were grouped into the following categories; demographics (age and marital status), household (separate kitchen, house type and number of rooms), exposure activity (hours in kitchen and tobacco use). Age was grouped into three categories (15–24, 25–34, 35–45) years. Also, variables for children were grouped into the following categories; demographics (age, sex, number of siblings, birth order), exposure activity (exposure to biomass smoke and exposure to tobacco smoke in the living room), and household (separate kitchen, house type and number of rooms). Age was grouped into four categories (0–6, 7–12, 13–24, 25–59) months. We defined a separate kitchen as a detached structure used for cooking that is outside the main living area of the house. 

We asked the question, ‘What type of biomass fuel does your household normally use for cooking?’, and this eventually allowed us to categorize wood and charcoal fuels separately. We defined ARI for the subjects as those who suffered from cough, followed by rapid breathing in the two weeks that preceded the survey. This definition was in agreement with existing literature [14,15]. We considered ones presence in the kitchen as risk factor for ARI, and every woman that was included in the survey met this criterion given that they cook every day. 

Primary health care workers (two community nurses), one community outreach health care worker, and two public health students served as field volunteers. Field volunteers orally translated the questionnaires into the three local dialects, namely Krio, Temne and Mende, during interviews for some subjects. 

### 2.2. SPM Monitoring

SPM was monitored using a digital realtime LD-3K2 dust monitor (Sibata Scientific Technology Ltd., Tokyo, Japan) equipped with a laser that measures relative concentration of respirable particles through the intensity of scattered light. Prior to monitoring each day, background and span checks were conducted to remove filled air in the detector, to compare the measured value accurately of the light scattering plate. The relative concentration of SPM with size less than 10 µm in diameter was measured every minute, and expressed as count per minute (CPM). A previous study has shown that optical count of particles correlates highly with gravimetric measurement of particles [21]. Thus, it is possible to calibrate light scattering particle counter against gravimetric measurement, and the precise factor to convert CPM to µg/m^3^ determined. In the absence of gravimetric measurement, however, particle count could be used as a close measure of SPM concentration. In this study, we report the relative concentration of SPM as CPM, and the description and conversion of CPM to mass concentration (µg/m^3^) has been described elsewhere [22]. We considered 1 CPM to be approximately 1.3 µg/m^3^ according to the official conversion K-value for indoor environments in Japan [23]. 

The level of SPM was monitored in fifteen out of sixteen selected homes during the 21 day survey, to ascertain the levels in homes that reported cases of ARI. One home was selected in each stratum for monitoring. As the first few days were assigned for field preparation, only sixteen homes were considered for monitoring. Prior information of potential ARI condition for subjects was obtained from field volunteers a day before monitoring, and selection of homes was based on the first that was interviewed each day. Data from the kitchen, living room and outdoor in eight homes with wood stoves and seven homes with charcoal stoves were obtained. We defined the living room as a place where biomass burning was absent. Monitoring was done at a height of 1 m in the three locations. In the kitchen, monitors were placed 1.5 m away from the cook stove which is considered a reasonable distance for human exposure. In the living room, monitors were placed in a location that would not obstruct daily activities of occupants. Outdoor monitoring was made 4–5 m from the kitchen location. 12-hours daytime monitoring was conducted in the kitchen and outdoor locations, and 24-hours in the living rooms. 

### 2.3. Data Analysis

We applied descriptive statistics to summarize information gathered on ARI cases and associated covariates. Pearson chi-square test was used to test for independence between the main explanatory variable (fuel type) against the others, and t-test was used to compare mean SPM concentration. Logistic regression was used to estimate the effect of wood relative to charcoal fuels on ARI, with effect size reported in terms of odds ratio at 95%CI. Adjusted variables in the analysis were age, marital status, kitchen type, tobacco use, housing type, and number of rooms for women; and age, sex, number of siblings, exposure to biomass smoke and exposure to tobacco smoke, separate kitchen, house type and number of rooms for children. Household variables, often regarded as socio-economic factors were adjusted for both subjects, to estimate the effect of fuel type on ARI prevalence. These analyses were conducted using SPSS version 20. 

### 2.4. Ethics

Written informed consent to participate in the study was obtained for each subject (women), who gave consent for the child. Participants were informed about the purpose of the study, and they were free not to participate in the study. The study was given ethical approval by the Ethical Committee of Yokohama National University, Japan. We also received a follow-up approval from Njala University Institutional Review Board. 

**Table 1:** Sample distribution of women age 15–45 years by fuel type and other variables.

	Homes with Wood Stove	Homes with Charcoal Stove	
**Characteristics**	**Sample Distribution No.**	**Sample Distribution No.**	***p*** **-value**
Total	236	284	
Age (years)			
15–24	92	110	
25–34	82	131	0.0025
35–45	62	43	
Marital Status			
Married	182	201	
Single	30	69	0.0014
Widowed	8	3	
Separated	16	11	
Hrs in kitchen			
1–3	91	171	
4–6	129	113	<0.001
≥7	16	0	
Separate kitchen			
Yes	227	188	<0.001
No	9	96	
Tobacco use			
Current smoker	26	13	
Former smoker	13	10	0.0093
Never smoke	197	261	
House type			
Cement	49	167	
Mud	175	104	<0.001
Metal Sheets	12	13	
#of rooms			
1	59	85	
2	46	81	0.0012
3	61	72	
≥4	70	46	

## 3. Results

The distribution of women age 15–45 years with regards to other variables is summarized in Table 1. Low *p*-values of Pearson chi-square test justify rejection of the null hypothesis that the main explanatory variable (fuel type) is independent of the other variables. For example, women in homes using charcoal stoves appeared to spend less time (1–3 hours) in the kitchen (60%) than those using wood stoves (39%), perhaps due to the fact charcoal is a more efficient fuel than wood. The results suggests that women using wood stoves prefer separate outdoor kitchens (96%) more than those using charcoal stoves (66%), probably due to the smoky nature of wood. It would appear that greater number of women in homes with charcoal stoves (59%) lived in homes made from cement than those in homes with wood stoves (21%). This observation could possibly be due to better socio-economic status of women in homes with charcoal stoves. 

**Table 2:** Sample distribution of children age 0–59 months by fuel type and other variables.

	Homes With Wood Stove	Homes with Charcoal Stove	
**Characteristics**	**Sample Distribution No.**	**Sample Distribution No.**	***p*** **-value**
Total	236	284	
Age (months)			
0–6	44	45	
7–12	36	57	0.3433
13–24	86	110	
25–59	70	72	
Sex			
Male	118	146	0.7491
Female	118	138	
No. of siblings			
1	54	97	
2	45	77	
3	37	50	<0.001
≥4	100	60	
Birth order			
1	55	98	
2	43	75	
3	40	50	<0.001
≥4	98	61	
Exposure to biomass			
smoke ^a^			
Yes	187	172	<0.001
No	49	112	
Environmental			
tobacco smoke ^b^			
Yes	57	51	0.0830
No	179	233	

^a^ Exposure to biomass smoke: includes children who are exposed to either wood or charcoal smoke in the kitchen; ^b^ Environmental tobacco smoke: children living in homes that have one or more individual(s) that smoke tobacco.

Table 2 shows how the sample of children is distributed among the variables included in the analysis. Focusing on highly significant variables, it would appear that children with three or more siblings are more likely to live in homes with wood stoves (42%) compared with those that use charcoal (21%). Such an observation would suggest that mothers using wood have little resources to purchase charcoal which is more expensive than wood. Children in homes with wood stove are more exposed to smoke in the kitchen (79%) than their counterparts in homes with charcoal stove (61%), and this gives an indication of the socio economic scale and its relationship with child caring.

**Table 3:** ARI prevalence in women (age 15–45 years) and the odds ratio estimate of the effect of cooking fuel types with adjusted variables during the two weeks preceding the survey.

	Homes with wood stove	Homes with charcoal stove				
Characteristics	ARI prevalence No. (%)	ARI prevalence No. (%)	OR (95%CI) *Unadjusted*	p-value	OR (95%CI) *Adjusted*	*p*-value
Fuel						
Wood	75 (31.7)	-	1.48 (1.01–2.17)	0.047	1.14 (0.71–1.82)	0.580
Charcoal *	-	68 (23.9)	1.00		1.00	
Age						
15–24 *	33 (35.8)	27 (24.5)	1.00		1.00	
25–34	25 (30.4)	34 (25.9)	0.91 (0.59–1.38)	0.652	0.75 (0.47–1.19)	0.220
35–45	17 (27.4)	7 (16.2)	0.70 (0.41–1.21)	0.203	0.40 (0.21–0.77)	0.006
Marital Status						
Married *	57 (31.3)	50 (24.8)	1.00		1.00	
Single	7 (23.3)	13 (18.8)	0.65 (0.38–1.11)	0.121	0.66 (0.37–1.18)	0.158
Widowed	3 (37.5)	1 (33.3)	1.47 (0.42–5.13)	0.543	1.73 (0.47–6.48)	0.407
Separated	8 (50.0)	4 (36.3)	2.06 (0.93–4.55)	0.073	1.35 (0.56–3.24)	0.499
Hrs in kitchen ‡						
1–3 *	26 (28.5)	38 (22.2)	1.00			
4–6	42 (32.5)	30 (26.5)	1.31 (0.88–1.94)	0.179		
≥7	7 (43.7)	0 (0)	2.40 (0.86–6.72)	0.094		
Separate						
kitchen	71 (31.2)	52 (27.6)	1.00		1.00	
Yes *	4 (44.4)	16 (16.6)	0.56 (0.32–0.94)	0.032	0.68 (0.38–1.24)	0.210
No						
Tobacco use						
Current smoker	15 (57.6)	7 (53.8)	4.04 (2.07–7.89)	<0.001	4.75 (2.28–9.90)	<0.001
Former smoker	7 (53.8)	3 (30.0)	2.40 (1.02–5.63)	0.043	3.37 (1.33–8.52)	0.010
Never smoke *	53 (26.9)	58 (22.2)	1.00		1.00	
House type						
Cement *	13 (26.5)	41 (24.5)	1.00		1.00	
Mud	60 (34.2)	27 (25.9)	1.35 (0.91–2.02)	0.131	0.96 (0.60–1.52)	0.849
Metal Sheets	2 (16.6)	0 (0)	0.26 (0.05–1.14)	0.075	0.23 (0.05–1.12)	0.069
#of rooms						
1 *	18 (30.5)	26 (30.5)	1.00		1.00	
2	14 (30.4)	16 (19.7)	0.70 (0.40–1.20)	0.202	0.65 (0.37–1.17)	0.153
3	18 (29.5)	13 (18.0)	0.69 (0.40–1.17)	0.176	0.64 (0.36–1.15)	0.135
≥4	25 (35.7)	13 (28.2)	1.10 (0.65–1.87)	0.704	1.07 (0.59–1.92)	0.816

* Reference group; ‡ Adjustment for time spent in kitchen was largely ignored in the analysis because the risk of ARI is likely to be related to cumulative exposure. *p-*value is significant at 0.05 for the associations.

**Table 4:** ARI prevalence in children (under 5 years) and odds ratio estimate of the effect of cooking fuel types (wood and charcoal) and other variables during the two weeks preceding the survey.

	Homes with wood stove	Homes with charcoal stove				
Characteristics	ARI prevalenceNo. (%)	ARI prevalenceNo. (%)	OR (95% CI)*Unadjusted*	*p*-value	OR (95% CI)*Adjusted*	*p*-value
Fuel						
Wood	150 (63.5)	-	2.25 (1.58–3.21)	<0.001	2.03 (1.31–3.13)	0.001
Charcoal *	-	124 (43.6)	1.00		1.00	
Age						
0–6 *	29 (65.9)	15 (33.3)	1.00		1.00	
7–12	27 (75.0)	27 (47.3)	1.41 (0.78–2.54)	0.244	1.63 (0.86–3.06)	0.133
13–24	54 (62.7)	58 (52.7)	1.36 (0.82–2.25)	0.227	1.53 (0.86–2.69)	0.141
25–59	40 (57.1)	24 (33.3)	0.83 (0.49–1.42)	0.517	0.87 (0.48–1.57)	0.653
Sex						
Male *	78 (66.1)	63 (43.1)	1.00		1.00	
Female	72 (61.0)	61 (44.2)	0.94 (0.66–1.33)	0.740	1.04 (0.72–1.49)	0.843
# of siblings						
1 *	36 (66.6)	43 (44.3)	1.00		1.00	
2	31 (68.8)	36 (46.7)	1.11 (0.68–1.79)	0.669	1.15 (0.69–1.91)	0.587
3	21 (56.7)	19 (38.0)	0.77 (0.45–1.31)	0.346	0.78 (0.44–1.37)	0.385
≥4	62 (62.0)	26 (43.3)	1.11 (0.71–1.74)	0.635	0.89 (0.55–1.45)	0.648
Birth order ‡						
1 *	37 (67.2)	44 (44.8)	1.00			
2	28 (65.1)	35 (46.6)	1.01 (0.62–1.64)	0.942		
3	25 (62.5)	19 (38.0)	0.85 (0.50–1.43)	0.542		
≥4	60 (61.2)	26 (42.6)	1.04 (0.67–1.63)	0.839		
Exposure to biomass						
smoke ^a^						
Yes	120 (64.1)	78 (45.3)	1.37 (0.94–1.99)	0.094	1.00 (0.65–1.55)	0.998
No *	30 (61.2)	46 (41.0)	1.00		1.00	
Environmental						
tobacco smoke ^b^						
Yes	39 (68.4)	25 (49.0)	1.39 (0.91–2.15)	0.126	1.39 (0.87–2.21)	0.163
No *	111 (62.0)	99 (42.4)	1.00		1.00	
Separate Kitchen †						
Yes*	146 (64.3)	91 (48.4)	1.00		1.00	
No	4 (44.4)	33 (34.4)	0.41 (0.26–0.63)	<0.001	0.54 (0.32–0.88)	0.014
Housing Type †						
Cement*	32 (65.3)	68 (40.7)	1.00		1.00	
Mud	113 (64.6)	50 (48.1)	1.63 (1.13–2.32)	0.007	1.11 (0.73–1.68)	0.621
Metal Sheets	5 (41.6)	6 (46.1)	0.91 (0.39–2.09)	0.827	0.63 (0.25–1.54)	0.309
# of rooms†						
1 *	37 (62.7)	43 (50.5)	1.00		1.00	
2	32 (69.5)	35 (43.2)	0.92 (0.57–1.48)	0.740	0.89 (0.54–1.49)	0.679
3	36 (59.0)	29 (40.2)	0.74 (0.46–1.19)	0.216	0.71 (0.42–1.17)	0.178
≥4	45 (64.3)	17 (36.9)	0.91 (0.56–1.50)	0.734	0.73 (0.42–1.25)	0.250

* Reference group; ^a^ Exposure to biomass smoke: includes children who are exposed to both wood and charcoal smoke in the kitchen; ^b^ Environmental tobacco smoke: children living in homes that have one or more individual(s) that smoke tobacco. *p*-value is significant at 0.05 for the associations; ‡ Birth order was excluded in the adjusted analysis for children because it correlates highly with number of siblings; † These variables were included in the adjusted analysis to account for household variables, as they were collected on the same form for women.

Table 3 contains a list of variables indicating ARI two-week period prevalence, the unadjusted and adjusted odds ratio estimate of the effect of cooking fuel types for each category of variable for women. We observed that 32% of ARI prevalence was in homes using wood stove compared with 24% in homes using charcoal stove. 

ARI prevalence appears to increase with increase in time spent in kitchens with wood stoves, but the increase is not as obvious in kitchens with charcoal stoves. The data suggests that ARI prevalence increase from non-smokers to current smokers by a factor of 2, regardless of the type of fuel. It thus seems that smoking would appear to increase the risk of developing ARI (current smoker *vs*. non smoker, (OR = 4.75; 95%CI: 2.28–9.90), former smoker *vs*. non smoker, (OR = 3.37; 95%CI: 1.33–8.52)). Women age 35–45 years somehow have lower ARI prevalence among the other age categories irrespective of the type of fuel. The unadjusted effect of wood smoke on ARI prevalence appears to be significant (OR = 1.48; 95%CI: 1.01–2.17). Adjusting for potential confounders indicated wood smoke not to be significantly associated with the risk of developing ARI (OR = 1.14; 95%CI: 0.71–1.82). 

Table 4 provides a summary of variables for ARI two-week prevalence, and the unadjusted and adjusted odds ratio estimate of the effect of fuel type for each category of variable for children. The reported ARI prevalence was 64% for children in homes with wood stoves compared with 44% in homes with charcoal stoves. Generally, ARI prevalence appeared higher for children in homes with wood stoves compared with children in homes with charcoal stoves for each variable. The effect of wood smoke is a significant risk factor to develop ARI from the unadjusted odds ratio (OR = 2.25; 95%CI: 1.58–3.21), and adjusted odds ratio (OR = 2.03; 95%CI: 1.31–3.13). Looking at the confidence interval for the adjusted data, separate kitchen is a significant risk factor of ARI for children (OR = 0.54; 95%CI: 0.32–0.88). All other variables do not seem to be significantly related to the risk of ARI. In terms of odds ratio, data would suggest that children age 7–12 months and 13–24 months have 63% and 53% higher risk of suffering from ARI compared with the reference group. These risks, however, are not significant at the 95% level, but are worth noting as they would become significant at the 85% significance level (*p*-values 0.133 and 0.141). In view of the results found for women, it is surprising that passive smoking is not a significant risk factor for children to develop ARI (OR = 1.39; 95%CI: 0.87–2.21) but children exposed to tobacco smoke are 39% more likely to suffer from ARI than children who are not exposed, but the risk would be significant at 80% significance level (*p* = 0.163). 

The ventilation of the household might be impacted by household level variables, such as kitchen type, house type and number of rooms. These variables are also considered as socio-economic indicators, and poorer households are generally more likely to use wood than charcoal. These variables are likely to confound the association between ARI and fuel type. Adjusting these variables for both women and children led to the same conclusion and statistical significance in Table 3 and Table 4. The odds ratio of the effect of wood smoke on ARI for women is (OR = 1.24; 95%CI: 0.78–1.95; *p*-value = 0.349) and (OR = 1.91; 95%CI: 1.26–2.89; *p*-value = 0.002) for children. 

Summary statistics of SPM concentrations in three locations for homes with wood and charcoal stoves are presented in Table 5. Mean SPM concentration for the different locations tend to decrease from the kitchen to living room to outdoor for homes using wood or charcoal stove. Kitchens with wood stove had significantly higher indoor concentration of SPM than charcoal stove. There was no difference between concentration of SPM in the living room of homes with wood and charcoal stoves. 

**Table 5:** SPM levels in count per minute (CPM) in three different locations.

Location	Homes with Wood Stoves	Homes with Charcoal Stoves	*p*-value
Kitchen [12-hour]			
n	8	7	
Mean	882.4	197.2	
Std. Dev	518.0	136.0	0.003
Minimum	302.0	29.0	
Maximum	1,635.0	398.7	
Living room			
[24-hour]			
n	7	6	
Mean	64.5	59.0	0.439
Std. Dev	25.4	74.0	
Minimum	23.1	15.0	
Maximum	100.1	206.0	
Outdoor [12-hour]			
n	8	5	
Mean	48.4	28.3	0.079
Std. Dev	27.0	20.0	
Minimum	23.1	11.1	
Maximum	111.0	61.4	

Square brackets indicate the sampling period. The difference in ‘n’ among the three locations was due to failure of the monitoring devices. In total, fifteen homes were monitored during the survey due to failure of the monitoring devices. 

## 4. Discussion

Acute respiratory infections (ARI) could represent a major health concern in Western Area of Sierra Leone. We found that women and children living in homes using wood for cooking had higher ARI prevalence than their counterparts using charcoal. The huge dependence on wood and charcoal fuels is an indicator of poor socio-economic status, which has been associated with deteriorating respiratory health, particularly for children [24,25,26]. Our results are well above the national average reported for children under five years in Sierra Leone [27]. The report indicated modest ARI prevalence (9%), but went further to emphasize that ARI prevalence peak in July and August, a period with the heaviest annual rainfall, which incidentally was almost the same period that this survey was conducted. The ARI prevalence in the current study was higher than previous studies reported [12,14]. It may be that communities where the present study was conducted greatly suffered from ARI, which also coincided with the peak period in Sierra Leone. Again these communities were not representative of the national population, which is in contrast to the referred studies. 

After adjusting for age, marital status, kitchen type, tobacco use, housing type, and number of rooms for women, the effect of wood smoke on ARI prevalence was similar to that of charcoal smoke, suggesting no significant health benefit in reducing the risk of ARI if charcoal is used. On the contrary, the effect of wood smoke on ARI prevalence for children remained large, after adjusting for age, sex, number of siblings, exposure to biomass smoke, exposure to tobacco smoke, separate kitchen, house type and number of rooms, indicating a significant health benefit in reducing the risk of ARI if charcoal is used. The contrast in health benefits for the two groups could be due to the risk factors used in the model. As children are more susceptible to the risk of ARI than women, partly due to their weak respiratory defense systems, and since the anatomy of their airways are immature [5], it means that the risk factors could strongly influence the effect of wood smoke on ARI in the adjusted model. Thus, the health benefits of ARI prevalence if charcoal is used should be considered in the light of the risk factors used in the model for the two groups. 

We have found at 85% significance level that children age 7–12 months and 13–24 months are more likely to suffer from ARI than children age 0–6 months. Children start crawling about this age bracket and they are likely to follow their mothers to the kitchen, thus increasing their exposure levels to biomass smoke. Also, they start supplementary feeding around 6 months, which potentially takes away the protection offered by breast feeding, and are likely to be fed with contaminated food supplements [14]. Children exposed to tobacco smoke are more likely to have suffered from ARI than those who are not exposed, although the association is not significant at 95% level (*p* = 0.163). We share similar observation with a previous study [28]. ARI prevalence was high even with the small proportion of children exposed to environmental tobacco smoke compared with a previous evidence [28]. A strong evidence from a meta analysis indicated that exposure to environmental tobacco smoke could influence respiratory health outcome in early childhood [29]. Although greater proportion of women did not smoke tobacco, yet, the small proportion of active and former smokers are more likely to suffer from ARI. This observation is in line with existing evidence that tobacco smoke is a risk factor of respiratory diseases. 

The information obtained on ARI was based on responses from women with no clinical diagnosis being made. Cough itself is a common symptom observed for other health conditions, but the symptomatic definition in the current study was meant to account for a reasonable estimate of acute lower respiratory infection (ALRI), although some acute upper respiratory infection (AURI) may have been reported. This may have led to misclassification of ALRI estimates. As obtaining clinical data under field conditions in Sierra Leone is impracticable or remote, it means an alternative field approach to identify the disease reasonably. Previous report suggest that the respiratory rate is a valuable clinical sign to diagnose ALRI especially in children who are coughing and breathing rapidly [30]. Because it is difficult to differentiate between ALRI and AURI, we used the term ARI in this study. 

Although the study drew attention to the impact of smoke generated from wood and charcoal stoves on ARI prevalence in a developing country, this study, like others, also suffer from drawbacks when considering potential policy implications. First, a cross sectional study does not often account for past exposure or recent changes in cooking methods. This could create difficulty in interpreting the results, because the risk factors and health outcomes are measured at the same time. As the risk of ARI is likely to be cumulative over time, the estimate of ARI associated with a given fuel may be misleading. Second, even though we collected information for some confounding variables, this study may have failed to include all of the confounding variables for the subjects, such as child birth weight, stunted or normal growth, nutritional status, immunization history, women’s education and their absence could affect the overall effect of fuel type on ARI. Third, translation of the questionnaire was standardized in the field, and it did not undergo any forward-backward translation which would have given field volunteers ample time to do pre-tests leading to validation. This action was considered a limitation. Furthermore, the lack of direct exposure assessment of indoor air pollutants generated from biomass smoke in all the households is considered a drawback, considering the existing evidence of the association between indoor air pollutants and ARI. 

The present study represents one of the first studies to compare the impact of cooking smoke from wood and charcoal fuels on ARI prevalence. Since the association between biomass smoke (including wood and charcoal) and ARI is causal [5], then preventing exposure to this risk factor would minimize the risk of ARI. Even though charcoal is relatively less polluting than wood, ARI prevalence associated with the former in the present study was high for both women and children when compared with high polluting fuels elsewhere [14]. The present study brought understanding to wood and charcoal related ARI issues in typical communities in Western Sierra Leone where the majority of people depend on these fuels. It was imperative to compare ARI prevalence associated with smoke derived from wood and charcoal stoves, to confirm the hypothesis that women and children exposed to wood smoke would have more ARI prevalence than those exposed to charcoal smoke. Although wood and charcoal are both biomass-derived, they generate different extents of emissions and are associated with different ARI prevalence, but which were both high. The present findings reiterate the need for a progress to cleaner fuels in Sierra Leone. 
